# CaSR as a Therapeutic Target and Tool in Human Dental Pulp: A Concise Review and Novel Hypothesis

**DOI:** 10.3290/j.ohpd.a42688

**Published:** 2020-07-04

**Authors:** Shaofeng An, Yan Gao, Yanhuo Chen, Dongjia Lin

**Affiliations:** a Associate Professor, Department of Operative Dentistry and Endodontics, Guanghua School of Stomatology, Hospital of Stomatology, Sun Yat-sen University, Guangzhou, Guangdong 510055, PR China. Designed and supervised this project; drafted and finalised the manuscript.; b Professor, Department of Operative Dentistry and Endodontics, Guanghua School of Stomatology, Hospital of Stomatology, Sun Yat-sen University, Guangzhou, Guangdong 510055, PR China. Designed this project and drafted the first manuscript.; c Master’s Student, Department of Operative Dentistry and Endodontics, Guanghua School of Stomatology, Hospital of Stomatology, Sun Yat-sen University, Guangzhou, Guangdong 510055, PR China. Helped to collect the data and draft the manuscript.; d Master’s Student, Department of Operative Dentistry and Endodontics, Guanghua School of Stomatology, Hospital of Stomatology, Sun Yat-sen University, Guangzhou, Guangdong 510055, PR China. Helped to collect the data and draft the manuscript.

**Keywords:** calcium sensing receptor, calcimimetics, human dental pulp cells, osteogenic differentiation

## Abstract

**Purpose:**

To review the essential characteristics of calcium sensing receptor (CaSR) and explore the hypothesis that elevated extracellular calcium ions (Ca^2+^) may affect the odontogenic/osteogenic differentiation and mineralisation of human dental pulp cells (hDPCs) through the CaSR signal.

**Materials and Methods:**

Based on a literature search of databases using different combinations of the key words and our previous researches, we gleaned the following important viewpoints.

**Results:**

The Ca^2+^ released from pulp capping materials plays an essential role in maintaining the viability and function of human dental pulp, and elevated extracellular Ca^2+^ concentrations can promote the odontogenic/osteogenic differentiation and mineralisation of hDPCs. Ca^2+^ is the primary physiological ligand of the CaSR, which has been reported to be widely expressed in a broad range of cells, including various osteoblast-like cell lines, osteoprogenitor cells, and mature osteoblasts. hDPCs consist of different subpopulations and have been shown to share phenotypical features with osteoblasts. Thus, we speculated that hDPCs also express CaSR and respond to extracellular Ca^2+^ via this receptor. Calcimimetics are indirect allosteric regulators of CaSR function and can increase the receptor’s sensitivity to ambient Ca^2+^.

**Conclusion:**

The local use of calcimimetics and calcium-based pulp capping materials could create an option for promoting the Ca^2+^ influx of hDPCs from the extracellular space via the CaSR. Such elevated Ca^2+^ concentrations could enhance the odontogenic/osteogenic differentiation and mineralisation of hDPCs and eventually improve the success rate of direct pulp capping treatments in patients suffering from accidental dental pulp exposure.

Accidental exposure of the dental pulp is a clinical reality that requires optimal treatment in a variety of clinical dental practices, such as the removal of dental caries, crown fractures and tooth preparation.^17^ Dental pulp tissue has been shown to harbour various populations of multi­potential stem/progenitor cells. The main type of cell linage is composed of human dental pulp cells (hDPCs), which have multipotential characteristics and have been regarded as potentially useful sources for the regeneration of dentine in certain circumstances.^21,38^ Thus, in permanent teeth that are diagnosed with normal pulp or reversible pulpitis, direct pulp capping is a feasible therapeutic method for preserving the vitality of exposed and potentially infected pulp.^10,16,17^ In such treatments, once the exposure has occurred, the tooth must be isolated from the saliva to prevent contamination, and then an appropriate pulp capping material is placed directly over the exposed pulp to induce tertiary dentine formation. In brief, the main objectives of direct pulp capping are the maintenance of the pulp’s vitality and function, the promotion of the formation of a tertiary dentinal bridge, and the minimisation of bacterial microleakage.^10,16^

Although various factors affect the treatment outcome of direct pulp capping, the selection of the most appropriate capping material is believed to be essential for achieving good clinical results. Therefore, many clinicians and researchers have performed numerous studies of pulp capping materials.^16^ Due to their long history of inducing dentinal bridge formation to promote successful healing following injury, products containing calcium hydroxide (Ca(OH)2) are currently widely used as direct pulp capping materials,^13,20^ and, as has been the case for several decades, calcium hydroxide remains the gold standard treatment against which new materials are tested.^45^

Calcium hydroxide can affect the pulp repair process through one or more of several mechanisms of action. In an aqueous solution, calcium hydroxide separates into calcium ions (Ca^2+^) and hydroxide ions, which results in pH increases and the release of Ca^2+^.^13^ The therapeutic effects of calcium hydroxide, such as antimicrobial activity and induction of repair via hard tissue formation, have been attributed to this ability to break down into Ca^2+^ and hydroxyl ions.^13^ Although the antimicrobial activity of calcium hydroxide is related to the release of hydroxyl ions in aqueous environments, numerous studies have demonstrated that the Ca^2+^ play a greater role in mineralisation than do the hydroxyl ions because the Ca^2+^ promote cellular migration and differentiation.^34,43,48,53,56^ Ca^2+^ released from the pulp capping materials react with carbonic gas from the pulp tissue to form calcium carbonate, which promotes dental pulp cell proliferation and differentiation, and thus contributes to the onset of mineralisation.^13,34,35,43^^,53^

Traditionally, different formulations of calcium hydroxide and its compounds have been used for direct pulp capping therapies. However, there are disadvantages associated with the use of calcium hydroxide materials, which include the presence of tunnels in the dentine bridge, high solubility in oral fluids, lack of adhesion and degradation after acid etching.^27^ In recent years, several materials, such as mineral trioxide aggregate (MTA), tri-calcium phosphate ceramics and polyphasic calcium phosphates (Poly-CaP), have been proposed as alternatives to Ca(OH)2-based materials.^8,29^ These different pulp capping materials exhibit different solubilities and can release various concentrations of Ca^2+^ that lead to different clinical outcomes. The concentrations of Ca^2+^ that are released from these materials vary considerably and range from 0.094 millimolar (mM) to 0.982 mM.^13,42^

Previous studies have demonstrated that, compared with Ca(OH)2-based materials, MTA can achieved good outcomes by promoting the proliferation and odontogenic/osteogenic differentiation of hDPCs and that this enhancement is related to the greater capacity of MTA for the continuous release of Ca^2+^.^40,42,53^ Rashid et al showed that Ca^2+^ level increased by 0.2–0.7 mM specifically modulated osteopontin and bone morphogenetic protein-2 levels during hDPCs mineralisation.^43^ However, the effects of levels of ionic calcium greater than these (eg, 0.982 mM) that may be released from pulp capping materials on the odontogenic/osteogenic differentiation of hDPCs have not been thoroughly investigated. Moreover, many observations that higher Ca^2+^ concentrations increase osteoblast activity and inhibit osteoclastogenesis have been reported.^55^ Thus, we hypothesised that elevated Ca^2+^ concentrations would influence the odontogenic/osteogenic properties of hDPCs and result in different clinical outcomes following dental pulp capping.

## MATERIALS AND METHODS

### Increased Ca^2+^ Levels Affect the Odontogenic/Osteogenic Differentiation of hDPCs

To verify the hypothesis that elevated extracellular Ca^2+^ affects the odontogenic/osteogenic differentiation of hDPCs, we investigated the effects of various Ca^2+^ concentrations (ranging from 1.8 to 16.2 mM) on the odontogenic/osteogenic differentiation and mineralisation of hDPCs *in vitro*. The results revealed a trend towards a downregulation of the expressions of type I collagen and runt-related transcription factor 2 (Runx2) mRNAs at elevated concentrations of Ca^2+^, whereas osteopontin and osteocalcin mRNA expression were significantly upregulated.^2^ Higher Ca^2+^ concentrations greatly increased mineralised matrix nodule formation.^1,2^ These results were confirmed by subsequent studies of other groups.^28^ The Ca^2+^ concentration of 5.4 mM seemed to be the ideal concentration for the differentiation and mineralisation of hDPCs. Recently, other researchers reported that extracellular Ca^2+^ could also accelerate the odontoblastic differentiation of hDPCs induced by MTA.^26^

Based on these results, we identified the importance of optimising the release of Ca^2+^ from dental pulp capping materials for achieving desirable clinical outcomes. However, as mentioned above, the range of Ca^2+^ concentrations that are released from the currently employed direct pulp capping materials is 0.094–0.982 mM,^13^ which is much lower than this optimal concentration. Hence, increasing such relatively low local Ca^2+^ concentrations may enable us to enhance Ca^2+^ influx to promote the odontogenic/osteogenic differentiation and mineralisation of hDPCs.

hDPCs are cell-type functionally analogous to osteoblasts, and share many of the same functional properties as are found in the latter.^19^ They can also proliferate and differentiate into odontoblasts (specialised hard-tissue-forming cells), and participate in dentine formation both physiologically and developmentally through detecting external stimuli, such as the alkaline environment inside dental pulp treated with pulp capping materials (ie, Ca(OH)2 or MTA).^3,25^ Various researches have confirmed that both hDPCs and odontoblasts express a diversity of channels sensing extracellular Ca^2+^ fluctuation, which comprise voltage-gated Ca^2+^ channels (VGCC), cation-permeable transient receptor potential channels, store-operated Ca^2+^ entry channels (SOCE), voltage-dependent Na^+^ channels, and calcium sensing receptors (CaSR), among others.^22^

### CaSR and Calcimimetics

Ca^2+^ has been shown to have a crucial role in the regulation of many critical cellular functions such as proliferation, differentiation and fluid secretion. Numerous studies have reported that many organisms express cell-surface sensors for extracellular Ca^2+^. Ca^2+^ is the primary physiological ligand of a G-protein-coupled receptor that is termed the CaSR.^5,54^ Substantial evidence has accumulated that the CaSR is primarily associated with the maintenance of calcium homeostasis and skeletal functions, and that it is detected in a variety of bone marrow-derived cells, including osteoblasts, stromal cells, monocytes–macrophages and osteoclast precursor cells.^7,57^^-^^59^ The identification of the CaSR makes two potentially substantial additions to the bone model: the support of the chemotaxis of osteoblast precursors to the site of resorption via calcium gradients, and the inhibition of osteoclasts via the elevation of Ca^2+^ concentrations, which closes the negative feedback loop.^55^ The study of Chang et al indicated that the CaSR is absolutely required for bone growth and mineralisation in response to high extracellular Ca^2+^ concentrations.^6^ Additionally, recent research has shown that the CaSR plays essential roles in tooth and dental alveolar bone formation.^51^ Previous *in vivo* data indicated that CaSR signalling in bone cells is a physiological regulator of both osteoblastogenesis and osteoclastogenesis, which suggests the potential therapeutic applications of targeting the CaSR in bone in the context of bone disorders that are characterised by excessive bone resorption or reduced bone formation.^37,54,55^

Previous research has shown that the CaSR plays essential roles in tooth and dental alveolar bone formation.^51^ However, compared with the identification of CaSR in bone cells, the CaSR expression in human dental pulp and hDPCs still remains unclear and controversial. Tada et al reported that hDPCs do not express the transcript of CaSR,^52^ but a recent study indicated that CaSR is expressed in rat dental pulp tissue at high levels and hDPCs in low levels.^33^ hDPCs consist of different subpopulations that include osteo/odontoblasts, fibroblasts and undifferentiated mesenchymal cells, among others. It can also differentiate into three different cell lineages: adipogenic, neurogenic, and osteo/odontogenic.^21,38^ Many studies have shown that hDPCs share phenotypical features with osteoblasts.^23^ In addition, CaSR has been deemed to be involved MTA-mediated osteogenic differentiation of hDPCs.^24^ Therefore, we speculated that hDPCs also express CaSR and can respond to extracellular Ca^2+^ through these receptors. Moreover, the expression of CaSR in human dental pulp tissue needs further elucidation.

Although Ca^2+^ themselves can be considered to be the main activator of the CaSR, there are numerous known direct (type I agonists) and indirect allosteric (type II agonists/calcimimetics) regulators of the functions of CaSR.^47^ Type I agonists directly activate the CaSR by binding to its extracellular domain and include calcium and other divalent and trivalent cations, spermine, aminoglycoside antibiotics and some polyvalent amino acids and peptides. Type II agonists are also called calcimimetics and are not strictly agonists but are rather positive allosteric modulators that increase the receptor’s sensitivity to ambient Ca^2+^ (or other type I agents), typically by binding within the receptor’s transmembrane region.^37,44,49^ Several calcimimetic agents have already been developed and include the following: first-generation compounds, including NPS R-567, NPS S-567, NPS R-568, NPS S-568 and KRN-568; and a second-generation compound, cinacalcet hydrochloride (cinacalcet HCl, also known as AMG 073, NPS 1493, Sensipar, and KRN 1493 in Asia), which is the only calcimimetic agent that is currently approved by the FDA.^11,32,36,55^

Calcimimetics are unable to potentiate the effects of extracellular Ca^2+^ adult human bone cells (osteoblasts or osteoclasts) in the absence of CaSR expression,^41,46^ but are effective in cells that express the CaSR.^18^ Calcimimetic agents might have potential beneficial effects on bone cells that express the CaSR.^30^ Several recent studies have reported that R- and S-568 significantly increase CaSR expression at the level of the membrane and increase intracellular Ca^2+^ concentrations, thus these compounds might actively contribute to the process of osteogenesis.^4,12^ In osteoblasts, high Ca^2+^ concentrations stimulate differentiation and mineralised nodule formation, and this response can be blocked by the CaSR inhibitor NPS 89636. In summary, CaSR has pivotal roles in osteogenesis and mineralisation that can be manipulated with calcimimetics and CaSR inhibitors.

## RESULTS

## Hypothesis

**Fig 1:**
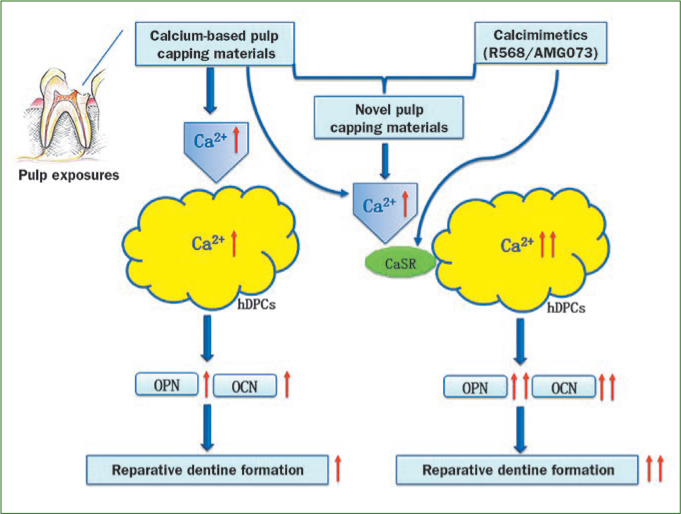
Diagrammatic representation of the hypothesis. Trauma would lead to accidental exposure of the dental pulp. Direct pulp capping with calcium-based pulp capping materials is a feasible therapeutic method. Elevated calcium ion (Ca^2+^) concentrations promote the odontogenic/osteogenic differentiation and mineralisation of human dental pulp cells (hDPCs). Ca^2+^ concentrations released from the currently available direct pulp capping materials is much lower. hDPCs may express the CaSR and respond to extracellular Ca^2+^ via this receptor. Calcimimetics can promote the Ca^2+^ influx of hDPCs from the extracellular space via CaSR. We make a hypothesis that the local use of calcimimetics and calcium-based pulp capping materials could create an option for enhancing the odontogenic/ osteogenic differentiation of hDPCs and improving the success rate of direct pulp capping treatments.

The elevation of Ca^2+^ concentrations and the use of calcimimetics strongly activate functional CaSR in several cell lines.^14,31,60^ Previous studies have strongly suggested that agonists that bind the CaSR may be advantageous in the treatment of bone injury.^9,12,30,50^ Therefore, according to our studies and the studies of other groups, we hypothesise that the local use of calcimimetics (ie, type II agonists), such as R-568 and/or AMG 073, allosteric modulators of the CaSR and calcium-based pulp capping materials, might be viable options for optimising Ca^2+^ release from dental pulp capping materials via the promotion of the influx of Ca^2+^ to hDPCs from the extracellular space via the CaSR. Such elevated Ca^2+^ concentrations would enhance the odontogenic/osteogenic differentiation and mineralisation of hDPCs and promote dentine bridge formation. Furthermore, the targeting of CaSR with calcimimetics and its inhibitors regulates the recruitment, differentiation and survival of osteoblasts and osteoclasts, which leads to bone cell metabolism and bone remodelling. This remodelling effect may help to prevent the pulp necrosis or calcification that is attributed to disturbances of the dental pulp blood circulation by absorbing the excessively formed dentine. Good blood circulation will benefit the maintenance of the vitality and function and aid the formation of normal dentine with a normal tubule structure. Finally, this approach might increase the success rate of direct pulp capping. The diagrammatic representation of this hypothesis is shown in Figure 1.

## Evaluation of the Hypothesis and Perspective

**Fig 2:**
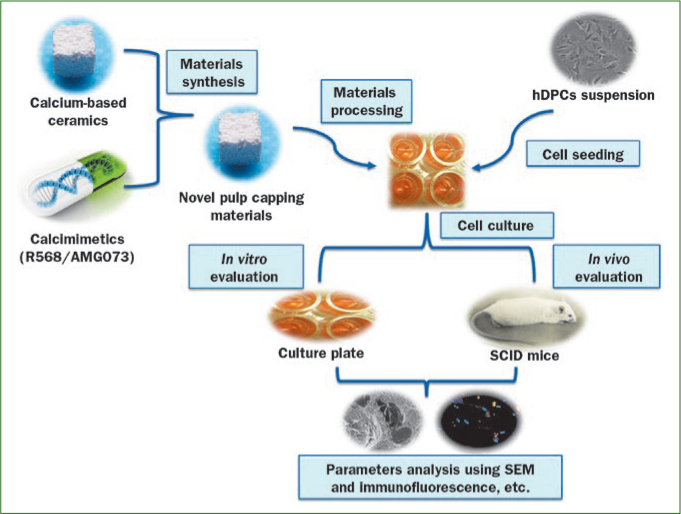
Evaluation of the hypothesis in three dimensional cultures. Novel pulp capping materials are developed by combination of calcium-containing ceramics and calcimimetics. hDPCs will be isolated and primary cultured from healthy and diseased tissues using explant culture method. Cells are seeded on scaffolds pretreated by specific culture medium. The cell-scaffold matrixes are cultured in basic or osteogenic inductive medium using microplates. Then a series of *in vitro* and *in vivo* experiments, such as immunohistochemical assay, scanning electron microscope, quantitative real-time polymerase chain reaction (PCR), Western Blot and animal experiments and so on should be undertaken to evaluate this hypothesis in the hDPCs.

First, hDPCs will be isolated and primary cultured from healthy and diseased tissues using explant culture method,^15,39^ and then a series of *in vitro* experiments should be undertaken to evaluate the expressions and localisations of CaSR mRNA and protein in the hDPCs and dental pulp tissue. Next, we need to select an appropriate calcimimetic agent based on tests of cell growth and apoptosis. The CaSR’s sensitivities to calcimimetics differ across cell types; the thyroid CaSR is 50–100-fold less sensitive to NPS R-568 than is the CaSR in the parathyroids.^49^ CaSR on osteoblasts might exhibit bidirectional control of osteoblast/osteoclast behaviour and potentially induce bone resorption in response to low Ca^2+^ concentrations.^55^ Hence the optimal combination of specific calcimimetics, Ca^2+^ concentrations, and the inductive microenvironments for hDPCs should be studied *in vitro*. Issues related to the efﬁcacies and safeties of such treatments will also require further animal investigation. Severely immunodeficient mice (SCID mice) and dogs will serve as good models for studying these issues. The evaluation method was illustrated in a proposed model presented in Figure 2.

## CONCLUSION

In summary, by lowering the threshold concentrations for Ca^2+^ or other cations-induced responses below the physiological levels of these nutrients in plasma membrane of hDPCs, CaSR could regulate key characteristics of the Ca^2+^ response at the single cell level, as well as the amplitude of whole tissue CaSR-mediated responses by recruiting quiescent cells into the active pool of responding cells. Ultimately, the confirmation of this hypothesis may result in a novel treatment that will improve the success rate of direct pulp capping in patients suffering from accidental dental pulp exposure.
